# Malacca leaf ethanolic extract (*Phyllanthus emblica*) as a hepatoprotector of the liver of mice (*Mus musculus*) infected with *Plasmodium berghei*

**DOI:** 10.14202/vetworld.2020.1457-1461

**Published:** 2020-07-27

**Authors:** Nuzul Asmilia, Dwinna Aliza, Yudha Fahrimal, Mahdi Abrar, Sulaiman Ashary

**Affiliations:** 1 Study Program Doctor Mathematical Applied Science, Universitas Syiah Kuala, Banda Aceh 23111, Indonesia; 2 Clinical Laboratory, Faculty of Veterinary Medicine, Universitas Syiah Kuala, Banda Aceh 23111, Indonesia; 3 Pathology Laboratory, Faculty of Veterinary Medicine, Universitas Syiah Kuala, Banda Aceh 23111, Indonesia; 4 Parasitology Laboratory, Faculty of Veterinary Medicine Universitas Syiah Kuala, Banda Aceh 23111, Indonesia; 5 Microbiology Laboratory, Faculty of Veterinary Medicine, Universitas Syiah Kuala, Banda Aceh 23111, Indonesia; 6 Veterinary Education Study Program, Faculty of Veterinary Medicine, Syiah Kuala University, Banda Aceh 23111, Indonesia

**Keywords:** hepatoprotector, Malacca leaf extract, megalocytosis, *Plasmodium berghei*

## Abstract

**Background and Aim:**

Although existing research confirms the antiparasitic effect of the Malacca plant against *Plasmodium*, its effect on the liver, one of the target organs of *Plasmodium* has not been investigated. Therefore, this study was conducted to explore the potential of the ethanolic extract of Malacca (*Phyllanthus emblica*) leaves in preventing liver damage in mice (*Mus musculus*) caused by *Plasmodium berghei* infection.

**Materials and Methods:**

This study was conducted using the livers of 18 mice fixed in 10% neutral-buffered formalin. A completely randomized design with a unidirectional pattern comprising six treatments was used in this study, with each treatment consisting of three replications. Treatment 0 was the negative control group infected with *P. berghei*, treatment 1 was the positive control group infected with *P. berghei* followed by chloroquine administration at a dose of 5 mg/kg BW, and treatments 2, 3, 4, and 5 were groups infected with *P. berghei* and administered Malacca leaf ethanolic extracts at doses of 100, 300, 600, and 1200 mg/kg BW, respectively. The extracts were administered orally using a gastric tube for 4 consecutive days. Mice were sacrificed on the 7^th^ day and livers were collected for histopathological examination.

**Results:**

Histopathological examination of the livers of mice infected with *P. berghei* demonstrated the presence of hemosiderin, hydropic degeneration, fat degeneration, necrosis, and megalocytosis. However, all these histopathological changes were reduced in the livers of *P. berghei*-infected mice treated with various doses of Malacca leaf ethanolic extract. The differences between the treatments were found be statistically significant (p<0.05).

**Conclusion:**

Ethanolic extract of Malacca leaves has the potential to protect against liver damage in mice infected with *P. berghei*. The dose of 600 mg/kg BW was found to be the most effective compared with the doses of 100, 300, and 1200 mg/kg BW.

## Introduction

Malaria is one of the deadliest diseases in the world. Although it can be prevented and treated, malaria impacts the health of people throughout the world. According to the World Health Organization (WHO), the majority of malaria cases reported in 2018 were from the WHO African region (213 million or 93%), followed by 3.4% of cases from the WHO Southeast Asian region and 2.1% of cases from the WHO Eastern Mediterranean region [1]. The annual parasite incidence of malaria in Indonesia was reported as 0.85 per 1000 population in 2015, with the highest number of cases being found in Papua [2]. It is known that malaria is caused by a protozoan parasite belonging to the genus *Plasmodium*, which consists of five major species causing malaria in humans, including *Plasmodium falciparum*, *Plasmodium vivax*, *Plasmodium malariae*, *Plasmodium ovale*, and *Plasmodium knowlesi*. Malaria in humans is generally transmitted through the bite of the female *Anopheles* mosquito infected with the parasites, whereas malaria in rodents is generally caused by *Plasmodium berghei* [3].

Most of the parasitic growth of *Plasmodium* in the host occurs in cells (intracellular), namely, the liver cells and red blood cells, thus causing anemia [4]. *P. berghei* infection may cause damage to important organs such as the lungs, liver, spleen, and brain [5]. The liver is the target organ of *Plasmodium* with an important role in the malaria cycle, acting as the site of parasitic activity and host immune response [6]. The liver is also a site for detoxification of toxins and drugs; therefore, it is important to protect the liver from the damaging effect of infectious agents and dangerous chemicals [7]. *P. berghei* infection results in histopathological changes in the liver such as sinusoidal dilatation, polymorphonuclear cells, hemosiderin, degeneration, hepatocyte necrosis, epithelial cell vacuolization, infiltration of mononuclear cells, and megalocytosis [4]. The management of *P. berghei* infection is highly dependent on chemical drugs; however, the resistance of the malarial parasite to available antimalarial drugs prompts researchers to determine more effective novel antimalarial drugs. In this context, research on medicinal plants that are used traditionally by communities in several places to treat malaria would be beneficial. Among such plants is the Malacca plant that has been reported to have antiparasitic and immune-enhancing properties and is also used for medicinal purposes and as a source of vitamins [8]. A previous study has demonstrated that the ethanolic extract of Malacca leaves inhibited the growth of *P. falciparum* at a concentration of 100 μg/ml, with an ability to inhibit 50% growth of *P. falciparum* at 3889 μg/ml [9]. The fruits, leaves, and roots of the Malacca tree contain polyphenols (tannins) and flavonoids [10]. Polyphenols are highly complex components of organic substances that can precipitate proteins from their solutions and combine with these proteins to repair damaged cells [11].

This study was conducted to determine the efficacy of the ethanolic extract of Malacca leaves (*Phyllanthus emblica*) in preventing liver damage in mice (*Mus musculus*) caused due to *P. berghei* infection.

## Materials and Methods

### Ethical approval

All experimental animal procedures were performed in compliance with the regulation of Animal Ethics Committee of Faculty of Veterinary Medicine, Universitas Syiah Kuala, Banda Aceh, Indonesia (Ref: 23/KEPH/III/2019).

### Study location and period

This study was conducted at Animal House Laboratory, Biomedical Development and Basic Technology of Health Centre, Health Ministry of Indonesia, Jakarta, and Pathology Laboratory, Faculty of Veterinary Medicine, Universitas Syiah Kuala. This study was conducted from March to April 2019.

### Experimental animals

A total of 18 male Balb/C mice, with an average body weight of 20-25 g and aged 2 months, were used in this study. *P. berghei* strain ANKA was obtained from the Animal House Laboratory, Biomedical Development and Basic Technology of Health Centre, Health Ministry of Indonesia, Jakarta.

### Experimental design

#### Plasmodium infection

A completely randomized design was applied in this study. The mice were randomly allocated to six groups (P0, P1, P2, P3, P4, and P5) treated with different doses of the leaf extract (modified from Jaijoy *et al.*, [12]). The treatments were performed with three replications. Treatment 1 (P0) was the negative control where the rats were only infected with *P. berghei*. In treatment 2 (P1), the rats were infected with *P. berghei* and then given chloroquine at 5 mg/kg BW. In treatments 3 (P2), 4 (P3), 5 (P4), and 6 (P5), the rats were infected with *P. berghei*, followed by the administration of the ethanolic extract of Malacca leaves at the doses of 100, 300, 600, and 1200 mg/kg body weight, respectively. All mice were injected intraperitoneally with 1 × 10^5^ *P. berghei* using a tuberculin syringe, and then the leaf extract was administered orally using a gastric sonde for 4 consecutive days. After 7 days, the mice were sacrificed and the livers were collected for histopathological examination.

### Histopathology preparations

The livers of mice were fixed in 10% neutral-buffered formalin solution for 24 h and dehydrated in an alcohol series. The clearing process was performed by submerging the liver samples in xylol solution. Subsequently, the infiltration step was conducted using liquid paraffin, followed by embedding the sample on the paraffin block. The paraffin-embedded samples were cut using a rotary microtome into 5- to 6-μm-thick sections. These sections were mounted on slides and then stained with hematoxylin and eosin [13]. Histopathological examination was conducted using a light microscope at a magnification of 10×40 in five visual fields (5 mm×5 mm). The following parameters were observed: Presence of hemosiderin, hydropic degeneration, fat degeneration, and liver cell necrosis.

### Statistical analysis

The obtained data were subjected to statistical analysis using analysis of variance, followed by Duncan’s test.

## Results

Histopathological examination of the livers of mice infected with *P. berghei* and then treated with the ethanolic extract of Malacca leaves demonstrated the presence of hemosiderin, hydropic degeneration, fat degeneration, necrosis, and megalocytosis. Table-1 shows the average number of mice liver cells that exhibited these histopathological changes.

**Table-1:** The average number of histopathological changes found in the liver of mice infected with *P. berghei* and treated with ethanol extract of Malacca leaves.

Treatments	Hemosiderin	Hydropic degeneration	Fat degeneration	Necrosis	Megalocytosis
P0	41.67±0.58^d^	27.00±0.00^e^	45.33±0.58^e^	26.67±0.58^d^	2.00±1.73^b^
P1	29.33±1.53^c^	22.33±0.58^d^	30.33±2.52^d^	21.33±4.04^c^	0.33±0.58^a^
P2	27.33±0.58^c^	14.67±0.58^c^	12.00±1.00^b^	11.33±1.53^b^	0.33±0.58^a^
P3	14.67±1.15^a^	11.67±1.15^b^	9.67±1.15^b^	5.00±0.00^a^	0.33±0.58^a^
P4	12.33±2.31^a^	6.33±0.58^a^	3.33±0.58^a^	4.00±0.00^a^	0.00±0.00^a^
P5	18.33±2.08^b^	13.67±2.52^bc^	16.00±1.00^c^	11.00±1.73^b^	0.00±0.00^a^

Different superscripts in the same column indicate highly significant differences (p<0.05). P0: Negative control (infected with *P. berghei* without the treatment of ethanolic extract of Malacca leaves); P1: Positive control (infected with *P. berghei* and administered chloroquine at 5 mg/kg BW); P2: Infected with *P. berghei* + ethanolic extract of Malacca leaves at dose 100 mg/kg BW; P3: Infected with *P. berghei* + ethanolic extract of Malacca leaves at dose 300 mg/kg BW; P4: Infected with *P. berghei* + ethanolic extract of Malacca leaves at dose 600 mg/kg BW; P5: Infected with *P. berghei* + ethanolic extract of Malacca leaves at dose 1200 mg/kg BW

Statistical analysis revealed significant differences in the average number of mice liver cells exhibiting the histopathological changes (hemosiderin, hydropic degeneration, fat degeneration, necrosis, and megalocytosis) between the treatments (p<0.05). The highest average number of liver cells with these changes was found in the P0 (negative control) group, whereas the lowest average number was observed in the P4 group (Table-1).

## Discussion

Microscopically, hemosiderin was found in all mice groups. Hemosiderin is a protein or blood amino acid that is formed when red blood cells are damaged [4]. Accumulation of hemosiderin in the liver tissue is caused due to the destruction of excessive red blood cells (hemolysis) due to the presence of parasites in the early stage infection leading to anemia in infected animals [14]. The damaged red blood cells release hemoglobin into the extracellular space. Phagocytic cells (from the mononuclear phagocyte system), also known as macrophages, ingest, or phagocyte hemoglobin, resulting in the production of hemosiderin and biliverdin [15]. A previous study also reported a similar result that *P. berghei* infection caused the accumulation of hemosiderin in mice liver [4].

**Figure-1:**
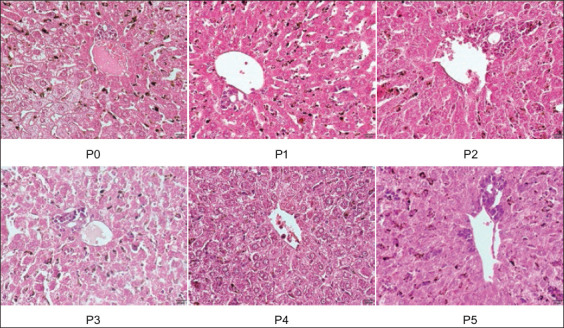
The histopathological feature liver of mice (*Mus musculus*) infected with *Plasmodium berghei* and treated with various doses of ethanolic extract of Malacca leave. (H&E, 400×).

Our study results demonstrated a decrease in the number of liver cells with hemosiderin in all treatment groups consistent with the increase in the administered dose (Figure-1). Group P4 mice administered 600 mg/kg BW of the ethanolic extract of Malacca leaves showed the lowest average number of liver cells with hemosiderin compared to that in other treatment groups. This result confirms that the terpenoids contained in the ethanolic extract of Malacca leaves inhibit the formation of hemosiderin. A previous study reported that artemisinin or terpenoids inhibited the production of biosynthetic hemosiderin, which was conducted in cell-free conditions at micromolar concentrations [16].

*P. berghei* causes cell injury such as hydropic and fat cell degeneration, which implies that the cell loses its normal structure before death [17]. Hydropic degeneration indicates a state of cytoplasmic cells containing water, whereas fat degeneration is a condition that describes hepatocytes containing lipids. This condition is caused due to disorders of liver metabolism in mice during *P. berghei* infection or toxic exposure, which results in reversible cell membrane damage. Damage to the cell membrane causes membrane leakage and disrupts the activity of K^+^ transport that exits the cell and the entry of Ca^2+^, Na^+^, and water into the cell. The entry of extracellular fluid into the cytoplasm causes swelling of the cytoplasm, mitochondria, and endoplasmic reticulum [18]. Moreover, the liver has an important role in regulating body fat, protein synthesis process, and phospholipids, thus inhibiting the synthesis and secretion of lipoproteins. Another study also demonstrated that *P. berghei* infection in mice caused hydropic degeneration and fat degeneration [19].

In the present study, the decrease in the average number of liver cells showing hydropic degeneration and fat degeneration in all treatment groups of mice was consistent with the increase in the dose of the ethanolic extract of Malacca leaves. Mice in the P4 group administered 600 mg/kg BW ethanolic extract of Malacca leaves exhibited the lowest average number of liver cells with both types of degeneration compared to that in other treatments. It appears that the effect of the extract to protect and recover the liver cells of mice infected with *P. berghei* is due to the antioxidant compounds contained in the extract, and one of them is triterpenoid. Triterpenoid compounds, as an antioxidant and scavenger against reactive oxygen species, can maintain the stability of the liver cell membrane [20].

Continuous degeneration can cause cell death known as necrosis, i.e., the death of cells or tissues in living organisms [21]. Necrosis in the liver may result from the direct influence of toxic agents such as chemicals and germ toxins [22]. High concentrations of chemicals in the liver lead to cell damage, such as infiltration of inflammatory cells, fat degeneration, and congestion [23]. Microscopically, the necrosis in hepatocytes is characterized by nuclear changes, i.e., absence of vascularization, loss of chromatin, wrinkles, dense and dark black in color (pycnosis), the nucleus is also torn and fragmented (karyorrhexis), and its color becomes pale and formless (karyolysis) [24]. Similarly, a previous study also reported the occurrence of cell necrosis in the liver of mice infected with *Plasmodium* [4].

The results of this study also demonstrated decreased number of necrotized liver cells in all treatment groups of mice according to the increases in the administered doses. Mice in group P4 administered 600 mg/kg BW of ethanolic extract of Malacca leaves showed the lowest average number of necrotic cells compared to that in mice from other treatment groups. The flavonoids present in the ethanolic extract of Malacca leaves acted as an antioxidant that inhibited the process of necrosis in liver cells, probably indicating the resolution of necrosis [25].

The presence of megalocytosis in the liver cells of mice was detected only in the control group (P0). Megalocytosis in the liver tissue occurs when red blood cells are infected with *Plasmodium* and is characterized by an abnormal enlargement of liver cells, i.e., megalocytosis. Megalocytosis also leads to the breakdown of red blood cells, which eventually causes bleeding and anemia in the infected animal [26,27].

Flavonoids are known to have hepatoprotective activity [28]. The leaf extract of *P. emblica* has been demonstrated to contain flavonoids [29]. Previous studies have reported that *P. emblica* fruit extract can also reduce the severity of hepatic damage [30-32]. Polyphenols and flavonoids exhibit high antioxidant and anti-inflammatory activities and thus protect against the development of oxidative stress [33]. Polyphenol compounds such as flavonoids inhibit oxidation reactions through the mechanism of radical scavenging by connecting one electron to unpaired electrons in free radicals so that the effects of free radicals are reduced [34]. It is believed that flavonoids have the ability to prevent liver damage by binding to free radicals causing impaired hepatocyte membrane integrity and escape of various enzymes from hepatocytes.

## Conclusion

Administration of the ethanolic extract of Malacca leaves was able to protect mice liver from the damage caused due to *P. berghei* infection, and the dose of 600 mg/kg BW was the most effective compared with the doses of 100, 300, and 1200 mg/kg BW.

## Authors’ Contribution

DA and NA designed the research. YF and NA performed *Plasmodium* infection. SA performed Histophatology. DA, NA, YF, and MA participated in drafting and revision of the manuscript. All authors have read and approved the final manuscript.

## References

[1] WHO (2018). World Malaria Report.

[2] Kemenkes RI (2016). Info Data Tingkat Malaria. PUSDATIN Kemenkes RI, Jakarta.

[3] Banyal N.A, Surianti A, Dayat R (2016). Klasifikasi citra plasmodium penyebab penyakit malaria dalam sel darah merah manusia dengan menggunakan metode multi class support vector machine (svm). J. Ilmiah Kompratif.

[4] Intan R.I, Lestari T.W, Sani Y (2017). Studi histopatologi pasca pemberian ekstrak campuran kulit batang pulai (*Alstonia scholaris*l R. Br.) dan meniran (*Phyllanthus niruri l*.) pada mencit terinfeksi *Plasmodium berghei*. J. Kedokteran Yarsi.

[5] Prasiwi D, Sudaryono A, Handayani D (2018). Aktivitas fraksi etanol dari ekstrak daun peronema carencens terhadap tingkat pertumbuhan *Plasmodium Berghei*. J. Pendidikan Dan Ilmu Kimia.

[6] Vanderberg J.P, Undra F (2004). Intravital microscopy demonstrating antibody-mediated immobilization of *Plasmodium berghei* sporozoites injected into skin by mosquitoes. Int. J. Parasitol.

[7] Wahyuningsih H.M.S, Mubarika S, Bolhui H.L.R, Nooter K, Wahyuono S (2002). Efek sitotoksik *in vitro* dari ekstrak daun mimba terhadap beberapa jenis lini sel kanker manusia. J. Kedokteran.

[8] Dhale D.A (2012). Pharmacognostic evaluation of *Phyllantus Emblica* Linn (*Euphorbiaceae*). Int. J. Pharma Bio. Sci.

[9] Asmilia N, Aliza D, Melia J, Rahmi E, Daulay L.S.M (2018). The effect of Malacca leaves (*Phyllantus Emblica*) ethanolic extract on *Plasmodium falciparum* growth *in vitro*. J. Kedok. Hewan.

[10] Mohanapriya M, Ramaswarny L (2012). Amla-the wonder of ayurvedic medicine. Int. J. Ayurved. Herb. Med.

[11] Teng C.W, Halin K.H, Ruwanaruk S, Hok L.K (2016). Medicinal Plants and Malaria:Applications, Trends, and Prospects. CRC Press, Boca Raton, USA.

[12] Jaijoy K, Soothornchareonnon N, Lertprasertsuke N, Panthong A, Sireeratawong S (2010). Acute and chronic oral toxicity of standardized water from fruit of *Phyllantus emblica* Linn. Int. J. Appl. Res. Natl Prod.

[13] Kiernan J.A (1990). Histological Histochemical Methods:Theory and Practice. Pergamon Press, Oxford, UK.

[14] Chandrasoma P (2005). Ringkasan Patologi Anatomi. EGC, Jakarta, Indonesia.

[15] Kalakonda A.J.S (2018). Fisiologi Bilirubin. EGC, Jakarta, Indonesia.

[16] Noeraini S, Fitri L.E.N (2004). Pengaruh ekstrak biji nimba (*Azadirachta Indica*) terhadap penurunan derajat parasite dan jumlah hemozoin terhadap kultur *Plasmodium falciparum*. J. Kedok. Brawijaya.

[17] Spector W.G, Spector T.D (2006). pengantar Patologi Umum. gadjah Mada University Press, Yogyakarta.

[18] King N.W, Joseph A (1996). Intracellular and Extracellular Deposition. Blackwell Publishing Professional, USA.

[19] Handani K.S, Utami W.S, Hermansyah B, Normasari R (2018). Gambaran histopatologis hati tikus Wistar pasca pemberian ekstrak etanol rimpang bangle pada uji toksisitas akut. J. Agromed.

[20] Bastona E, Leroux F.R (2007). Inhibitors of steroidal cytochrome p450 enzymes as targets for drug development. Recent Pat. Anticancer Drug Discov.

[21] Underwood J.C.E (1999). General and Systemic Pathology. International Publishing Leyden, Churchill Livingstone, United Kingdom.

[22] Ressang A.A (1984). patologi Khusus Veteriner. percetakan Bali, Denpasar, Indonesia.

[23] Guyton A.C, Hall J.E (2007). Buku Ajar Fisiologi Kedokteran.

[24] Himawan S (1992). Patologi. Penerbit Buku Kedokteran EGC, Jakarta, Indonesia.

[25] Annisya R (2011). Pengaruh pemberian ekstrak biji buah jamblang (*Syzigium cumini*) terhadap jumlah penurunan nekrosis dan apoptosis pada tikus (*Rattus novergicus*) yang terinduksi isoniazid. J. Kedok. UNS.

[26] Dkhill M (2009). Apoptotic changes induced in mice splenic tissue due to malaria infection. J. Microbiol. Immunol. Infect.

[27] Rusmiati L.A (2004). Struktur histologis organ hepar dan ren mencit (*Mus musculus*l) jantan setelah perlakuan dengan ekstrak kayu secang (*caesalpinia sappan*l). Bioscientiae.

[28] Alghazeer R, Elgahmasi S, Elnfati A.H, Elhensheri M, Al-Griw M.A, Awayn N, El-Nami M (2018). Antioxidant activity and hepatoprotective potential of flavonoids from *Arbutus pavarii* against CCl4 induced hepatic damage. Biotechnol. J. Int.

[29] Asmilia N, Abrar M, Fahrimal Y, Sutriana A, Husna Y (2020). Potential of Malacca leaf (*Phyllanthus emblica*) against *Salmonella* sp. E3S Web Conf.

[30] Lee C.Y, Peng W.H, Cheng H.Y, Chen F.N, Lai M.T, Chiu T.H (2006). Hepatoprotective effect of *Phyllanthus* in Taiwan on acute liver damage induced by carbon tetrachloride. Am. J. Chin. Med.

[31] Malar V, Mettilda M (2009). Hepato-protective activity o *Phyllanthus emblica* against paracetamol induced hepatic damage in Wister albino rats. Afr. J. Basic Appl. Sci.

[32] Huang C.Z, Tung Y.T, Hsia S.M, Wu C.H, Yen G.C (2017). The hepatoprotective effect of *Phyllanthus emblica* L. fruit on high fat diet-induced non-alcoholic fatty liver disease (NAFLD) in SD rats. Food Func.

[33] Pokorni J, Yanishileva N, Gordon M (2001). Antioxidant in Food Practical Application.

[34] Praman S, Mulvany M.J, Williams D.E, Andersen R.J, Jansakul C (2013). Crude extract and purified components isolated from the stems of *Tinospora crispa* exhibit positive inotropic effects on the isolated left atrium of rats. J. Ethnopharmacol.

